# Strength Is Still a Weakness in Coalition Formation: Replicating and Understanding the Strength-Is-Weakness Effect

**DOI:** 10.1177/01461672211005883

**Published:** 2021-04-13

**Authors:** Joeri Wissink, Ilja van Beest, Tila Pronk, Niels van de Ven

**Affiliations:** 1 Tilburg University, The Netherlands

**Keywords:** coalition formation, Strength-is-Weakness, replication, equity, online synchronous games

## Abstract

A key observation in coalition formation is that bargainers with most resources are often excluded from coalitions: the Strength-is-Weakness effect. Previous studies have suffered from low sample sizes and lack of (appropriate) incentives and have rarely focused on underlying processes. To address these issues, we conducted a cross-platform replication using the Online Coalition Game. We replicated the Strength-is-Weakness effect in a psychology laboratory, on Amazon Mechanical Turk, and on Prolific. Moreover, our results showed that the equity norm shapes the Strength-is-Weakness effect in two ways. First, strong bargainers claim a higher larger of the payoffs than weak bargainers do, making them less attractive coalition partners. Second, weak bargainers expect strong bargainers to make these larger claims, directing weak bargainers to each other from the outset. Finally, the studies suggest that the Online Coalition Game is a viable tool for conducting high-powered coalition formation research.

When goals cannot be attained by single individuals or parties, people often form coalitions. A formal definition of coalition formation is a situation in which there are more than two individuals or parties of which a subset needs to combine their resources to attain shared payoffs that are subsequently distributed among the members of the formed coalition (Gamson, 1964). For example, political parties combine their seats (resources) to obtain a majority in parliament to form a government and distribute the ministerial posts (payoffs). A striking observation is that coalition bargainers with the most resources are often excluded from coalitions, receiving no share of the payoffs at all; an observation dubbed the Strength-is-Weakness effect (Caplow, 1956; Chaney & Vinacke, 1960; Kelley & Arrowood, 1960; Murnighan, 1978a; van Beest et al., 2004b, 2011; Vinacke, 1959; Vinacke & Arkoff, 1957). Despite this widespread exclusion of those with a seemingly beneficial bargaining position—such as political parties with many seats in governmental coalition formation (Bäck & Dumont, 2008; Warwick, 1996)—both a high-powered replication of the effect and an investigation into the underlying mechanism has been thus far lacking.

In this article, we present three studies. Studies 1 and 2 are preregistered,^1^ piece-rate incentivized replications of the Strength-is-Weakness effect, one conducted in a standard social psychology lab setting and one on the online labor market Amazon Mechanical Turk, the first implementations of the novel Online Coalition Game: a tool for conducting (online) three-player interactive coalition experiments. Moreover, in these two studies and a third study, we focus on an alleged mechanism behind the Strength-is-Weakness effect: the (expected) use of the equity norm.

## The Strength-Is-Weakness Effect

A first mention of a Strength-is-Weakness effect is found in Caplow’s (1956) theorizing on coalitions in the triad. Caplow theorized that members of a triad may differ in strength and that strong members try to dominate weaker members. However, when the combined strength of the two weaker members would be sufficient to control the strongest member, the two weak members would form a coalition against the strong member.

Empirical evidence for the Strength-is-Weakness effect was first obtained using modified pachisi games (Chaney & Vinacke, 1960; Kelley & Arrowood, 1960; Vinacke & Arkoff, 1957). In these games, participants were part of a triad in which participants’ resources were represented by a *weight*: one participant (Player A) had a weight of 3, the other two (Players B and C) both had a weight of 2. Participants would receive a monetary payoff upon reaching the last space of a pachisi board. Each turn, a die was rolled and participants’ pawns moved the amount of pips on the die multiplied by their weight. Individually, Player A, having a higher weight, would always win. However, if two players would agree on how to distribute the payoffs among themselves if one of them reached the final space, these two players would add their weights together. In these experiments, the individually weak Players B and C often formed a coalition, thereby excluding the individually strong Player A. Player A was included in 28.9% of the cases, versus inclusion rates of 86.5% and 85.4% of Player B and Player C, respectively (Vinacke & Arkoff, 1957).

Further support for the Strength-is-Weakness effect has been found in simple weighted majority games (Komorita, 1984; Komorita & Parks, 1995) in which having more resources within a coalition does not bring additional benefit to the individual or coalition (Gamson, 1961b; Murnighan, 1978a; van Beest et al., 2004b, 2011). These situations are *simple* (Komorita, 1984), meaning that the payoffs are the same for every coalition, regardless of the combined resources. Moreover, in these situations, resources are *power-irrelevant* (Kravitz, 1981), meaning that all individuals have an equal number of winning coalitions that they are part of, regardless of their resources. Those with many resources thus do not have more bargaining opportunities—often referred to as *bargaining power*—than those with fewer resources. To be consistent with previous literature, we will nonetheless refer to them as *strong* and *weak* bargainers.

A common simple weighted majority game is the 5(432) game in which a coalition needs at least five resources to attain payoffs and in which every possible combination of bargainers with four, three, and two resources, respectively, for Players A, B, and C reaches this threshold (Murnighan, 1991). In this situation, a coalition between the weakest members (BC-coalition with five resources) is formed most often. Note that the Strength-is-Weakness effect has been found in simple weighted majority games situated in several kinds of simulated settings such as a political setting (Chertkoff, 1966), bargaining between company stockholders (Chertkoff & Braden, 1974), and negotiations concerning the joint sale of parcels of land (van Beest et al., 2004b).

## Reasons for Replication

### Lack of studies with strong evidential value

There are several reasons to suppose that the evidential value of previous studies on the Strength-is-Weakness effect is relatively low. As a general point, previous replication attempts of various effects in psychology have yielded low replication rates. For example, a large-scale replication attempt, which included 100 studies from three high-impact psychology journals, replicated only 39% of the effects (Open Science Collaboration, 2015). A possible contributor to false positives are small sample sizes, which increase the risk of false positives due to the (unconscious) use of researcher degrees of freedom (Ioannidis, 2005; Simmons et al., 2011). In coalition formation studies, more than two participants are inherently needed to assess which coalitions form. As the traditional laboratory has a limited pool of participants, it has often been difficult to reach an adequate sample size to ensure sufficient statistical power. Although this problem may be alleviated by letting participants complete multiple trials to attain more observations, this solution may lead to order and learning effects. For example, Kelley and Arrowood (1960) show quite substantial changes in formed coalitions in a 5(432) game after 10 to 70 trials. In these situations, it is difficult to determine which observations are valid and which have been transformed by repeated exposure and feedback.

Besides low sample sizes, previous studies often suffered from procedural issues that make us question their evidential value. One of these issues has to do with the necessity of experimenter–participant interaction in older studies in which offers slips were physically collected by the experimenter (e.g., Miller & Wong, 1986), possibly leading to experimenter bias. A second issue has to do with a lack of an adequate incentive structure. In many prior studies, participants often negotiated about hypothetical payoffs (Chaney & Vinacke, 1960; Kelley & Arrowood, 1960; Vinacke, 1959; Vinacke & Arkoff, 1957). This might be problematic in light of findings suggesting that a lack of incentives may lead to thoughtless responses from unmotivated participants (Camerer & Hogarth, 1999; Smith & Walker, 1993). It might be that when there is nothing at stake, a portion of strong bargainers in nonincentivized studies may have been unmotivated and applied a quick heuristic such as equity, which—as will be argued in the following section—is proposed to promote the exclusion of strong bargainers. If so, an incentivized experiment is a more conservative test of the Strength-is-Weakness. Other coalition formation experiments used tournament incentives, in which participants were reimbursed based on their performance relative to their peers (Murnighan, 1978b; van Beest et al., 2004b). This approach may be problematic because individuals become more risk-seeking under tournament incentives (Schedlinsky et al., 2016). In coalition formation settings, tournaments might incentivize participants to make risky offers in an attempt to maximize their payoffs. Hence, tournament incentives might inflate the Strength-is-Weakness effect, making the use of piece-rate incentives a more conservative test of the Strength-is-Weakness effect.

To address the abovementioned issues, we developed the Online Coalition Game using oTree (Chen et al., 2016), an open-source platform for behavioral research. The latest version of the Online Coalition Game, including a comprehensive wiki can be found here: https://github.com/JoeriWissink/OnlineCoalitionGame, with a further description at Wissink et al. (2021). The Online Coalition Game addressed the abovementioned issues as follows. First, the integration between oTree (Chen et al., 2016) and Amazon Mechanical Turk helped us obtain a substantial sample size while retaining an interactive design without the necessity of multiple trials. Second, we eliminated experimenter intervention by automatizing random matching of and interaction between participants. Third, we presented participants with a more straightforward piece-rate incentive scheme in which there was a fixed conversion rate between money earned in the experiment and an actual monetary bonus. Finally, to ward against research degrees of freedom, we made a data package including (meta) data, analysis scripts, stimulus materials, and preregistrations available on the Dutch Dataverse network: https://doi.org/10.34894/JXRELG. Moreover, in all studies, we report all measures and manipulations. No participants were excluded.

### Lack of understanding about underlying mechanisms

A second reason for conducting the studies in this article is that we want to understand the mechanisms behind the effect better; something we think is only possible in a study with strong evidential value. Previous literature has suggested three possible mechanisms.

#### The confusion hypothesis

According to the confusion hypothesis, the Strength-is-Weakness effect emerges because strong bargainers incorrectly equate their position of having more resource with a position of increased bargaining power. According to Vinacke and Arkoff (1957), strong bargainers’ unique position—they always win the game when no coalition is formed—make them slower than weak bargainers to realize that they need to secure a coalition before the other two bargainers do. A similar argument is brought forward by Kelley and Arrowood (1960) who claim that the Strength-is-Weakness is an artifact of the experiment used by Vinacke and Arkoff (1957) in which in some trials a weak coalition would not be strong enough to defeat the strong bargainers. According to Kelley and Arrowood, this could lead strong bargainers to perceive a correlation between resources and power, even in trials in which this was absent.

The confusion hypothesis has been extensively addressed in prior research. First, studies have shown that strong bargainers do not seem to believe that resources lead to more bargaining power (Wilke, 1968; Wilke & Mulder, 1971, 1974) and that explicit instructions that this is not the case do not prevent the Strength-is-Weakness effect (Vinacke et al., 1964). Second, the confusion hypothesis is only a tenable explanation for the effect in pachisi games, in which strong bargainers win if no coalition is formed. In simple weighted majority games, strong bargainers can only secure a share of the payoffs when in a coalition, making it highly unlikely that the Strength-is-Weakness effect observed in these games is due to misperceptions regarding resources and bargaining power. This is especially the case for experiments using the Komorita and Meek (1978) display protocol, in which all participants make opening offers at the same time, meaning that differences in who initiates the bargaining are eliminated as a possible cause of the effect.

#### Conspiracy theory

A second hypothesized reason for the Strength-is-Weakness effect places the causes for the effect in the hands of the weak bargainer. According to the conspiracy hypothesis (Wilke & Mulder, 1971, 1974), weak bargainers form a coalition in pachisi games because they believe it equalizes an unfair advantage handed to strong bargainers (i.e., strong bargainers win when no coalition is formed). There are two reasons why we find this account implausible. First, we do not think that forming a coalition against the strong bargainer would equalize the situation, but it actually puts the strong bargainer at a disadvantage. Second, if viable at all, this explanation would only explain Strength-is-Weakness effects in settings where strong bargainers have a higher chance (compared with weak bargainers) of obtaining payoffs without forming a coalition. Conspiracy theory is not a viable explanation in simple weighted majority games in which strong bargainers cannot single-handedly win and thus cannot be said to have an advantage to equalize by means of a conspiracy.

#### Use of the equity norm

A third, more promising, mechanism underlying the Strength-is-Weakness effect, is the distributive fairness concept of equity: the belief that someone’s payoff from a situation is fair when it is proportional to their input (Adams, 1965; Walster et al., 1973). Two classic coalition formation theories, minimum resource theory (Gamson, 1961a, 1964) and bargaining theory (Komorita & Chertkoff, 1973), implement this notion of equity by positing that coalition bargainers bargain for a payoff that is proportional to their resources in a coalition. This use of the equity norm means that strong bargainers demand a higher share of the payoffs than weak bargainers, thereby steering weak bargainers to form the *cheapest winning coalition*, in which their relative input—and thus expected share of the output—is highest. To illustrate this, let us consider a 5(432) situation in which Bargainers A (4 resources), B (3 resources), and C (2 resources) can allocate US$100 between the members of a coalition. Using the equity norm, A would rather form a coalition with C—in which they have 2/3 of the resources and thus expect to obtain about US$66—than a coalition with B in which they have only 4/7 of the resources and expect to obtain around US$57. Likewise, B and C would rather form a coalition with one another in which they have 3/5 and 2/5 of the resources and expect US$60 and US$40 out of the coalition, respectively, than only about US$43 or US$33 in a coalition with A.^2^ A consequence of these reciprocal first offers is that the BC-coalition will be formed most often, meaning that the strong bargainer is often excluded: the Strength-is-Weakness effect.

Although the equity norm plays a central role in classic coalition theories, previous studies in which a Strength-is-Weakness effect has been observed often lack detailed information on the implementation of equity. A few studies hint at the use of the equity norm in distribution of payoffs (Chertkoff, 1966; Chertkoff & Braden, 1974; Vinacke & Arkoff, 1957; Wilke & Mulder, 1974). Other studies hint at the expectation that other bargainers use the equity norm and/or resulting avoidance of strong bargainers (Chertkoff & Braden, 1974; Nitz & Phillips, 1969; van Beest et al., 2011; Wilke & Mulder, 1974). Surprisingly, no previous study in which a Strength-is-Weakness effect has been found reports enough information to deduce whether strong bargainers actually claim a higher share of the payoffs than their weaker counterparts do.

#### Remaining questions

Based on previous findings, the use of the equity norm seems the most plausible mechanism underlying the Strength-is-Weakness effect. As mentioned above, the confusion hypotheses and conspiracy theory receive little empirical and logical support. Conversely, the abovementioned findings suggest that the Strength-is-Weakness effect is due to adherence to the equity norm. The exact influence of the equity norm, however, remains unclear. As stated above, there is some evidence that coalition bargainers *expect* the use of the equity norm from other bargainers, which provides a rationale for a weak bargainer to approach the other weak bargainer. This evidence, however, is scarce and obtained in studies with small samples or without an adequate incentive structure. A second way the equity norm can contribute to the Strength-is-Weakness effect is when this norm is *applied* by bargainers: If strong bargainers make less attractive offers than weak bargainers do in their first offer, seeing this first offer will most likely lead to the formation of weak coalitions. Surprisingly, inquiries into these first offers have thus far been lacking in studies finding a Strength-is-Weakness effect.

In our three studies, we investigated both ways the equity norm can shape the Strength-is-Weakness effect. In all studies, we analyze first offers made by bargainers to investigate whether bargainers apply the equity norm: Do strong bargainers make more demanding initial offers than weak bargainers do? In Studies 1 and 2, we also analyze the effect of these first offers on formed coalitions by looking at whether the relative attractiveness of offers increases the likelihood of inclusion in a coalition. Moreover, we investigate whether the use of the equity norm is expected by bargainers by looking at whom they approach in their first offers (all studies) and by directly asking them which first and final offers they expect the other bargainers to make (Study 3). Finally, in our studies we utilize a simple weighted majority game, rather than a pachisi game, to rule out that an observed Strength-is-Weakness effect is due to confusion or conspiracy.

Studying the role of the equity norm in shaping the Strength-is-Weakness effect—and contrasting it against the other proposed mechanisms—helps us further our understanding of the Strength-is-Weakness effect in two ways. First, it helps elucidate whether the exclusion of strong bargainers is indeed driven by calculated and more or less rational attempts to maximize payoffs, rather than behavior guided by misperceptions (i.e., the confusion hypothesis) or by a more emotional response to perceived injustices (i.e., conspiracy theory). Second, further investigation concerning the equity norm will elucidate which bargainers are responsible for the Strength-is-Weakness effect. Evidence for the application of the equity norm will indicate that strong bargainers are (partially) responsible for the effect: by asking for an equitable share of the payoffs, strong bargainers will drive weak bargainers away from them, which would foster the formation of weak coalitions. Evidence for the expected use of the equity norm by weak bargainers would indicate that weak bargainers are (partially) responsible by the effect: expecting strong bargainers to use the equity norm, weak bargainers would avoid strong bargainers from the outset, promoting the formation of weak coalitions.

We also think that it is important to understand which mechanisms underlie the Strength-is-Weakness effect is because it helps bridge the gap between bodies of literature. On the one side, there is literature on the psychological consequences and underpinnings of interpersonal behavior that are theoretically rich, but often focus on the dyad or individual as a unit of measurement, such as literature on ostracism (e.g., Williams, 2007), perceived (bargaining) power (e.g., Handgraaf et al., 2008; Pinkley et al., 2019), and distributive preferences (e.g., Cappelen et al., 2007; Deutsch, 1975; van Dijk et al., 2004). On the other side, coalition formation provides a richer social context by taking into the effects of multiple interaction partners and the resulting questions regarding partner choice and making oneself an attractive coalition partner, but often lacks theoretical coherence. Understanding which psychological mechanisms underlie the Strength-is-Weakness—and coalition formation in general—will enrichen our understanding of coalition formation as well as cast a light on how certain psychological mechanisms operate beyond the scope of dyadic interaction.

## Study 1

The primary goal of Study 1 was to replicate the Strength-is-Weakness effect; the observation that strong bargainers are disproportionally often excluded from coalitions compared with their weaker counterparts. For this purpose, triads of students bargained in our social psychology lab in a 5(434) simple weighted majority game, using the Online Coalition Game. We expected to replicate the Strength-is-Weakness effect: that the weak coalition (BC-coalition with five resources) would be formed more often than both coalitions including the strong bargainer (AB-coalition with seven resources and AC-coalition with six resources).^3^

Moreover, we hypothesized that bargainers would both apply the equity norm and expect others to apply it. First, we expected that, as a result of applying the equity norm, strong bargainers demanded a higher share of the payoffs in their first offers than weak bargainers did. Moreover, we explored whether the relative attractiveness of these first offers predicted the formation of small coalitions (i.e., the Strength-is-Weakness effect). Second, expecting better offers from other weak bargainers, we predicted that weak bargainers would make their first offers to each other more often than to strong bargainers. In Study 3, we tested this expectation of equitable offers more directly by asking bargainers about the expected first and final offers from the other bargainers. Finally, we explored whether in formed coalitions strong bargainers acquired a higher proportion of the payoffs than weak bargainers did.

### Method

#### Participants and design

We recruited 180 undergraduate psychology students to take part in a study in our lab (*M*_age_ = 19.34 years, age range = 17–28, 142 females, 37 males, one other). Of these 180, we could eventually group 156 respondents into 52 triads. Participants were randomly assigned to one of three positions in a 5(432) landowner game: Landowner A with four resources, Landowner B with three resources, and Landowner C with two resources. The 24 participants who could not be matched did make a first offer and were paid according to how much they allocated to themselves in that first offer. As interpretations of results were the same regardless of including or excluding these 24 observations, we included them in the analyses that pertained to participants’ first offers.

Studies in our lab run for a maximum of 2 weeks, which served as our natural stopping rule. A sensitivity power analysis conducted with G*Power (Faul et al., 2007) revealed that we could detect a medium to large effect size (*w* = 0.43) when testing whether the distribution of formed coalitions differed from chance (i.e., equal proportions for all possible coalitions) with 80% power.

### Materials and Procedure

#### Game structure

Participants interacted through the Online Coalition Game (Wissink et al., 2021); an oTree version (Chen et al., 2016) of the landowner paradigm (van Beest et al., 2003), a contextualized simple weighted majority game. Participants took on the position of one of three landowners that each owned an unused parcel of land: Landowner A owned 4 acres of land, B owned 3 acres, and C owned 2 acres. A project developer offered to buy at least 5 acres of land for €100,000 and any coalition of two landowners could sell their parcels of land for this price. A coalition formed when two participants reached a consensus on how to distribute the €100,000 between the coalition partners. Participants received a €0.10 bonus for each €1,000 they gained.

#### Bargaining procedure

We adapted the Komorita and Meek display procedure, which consisted of three phases (Komorita & Meek, 1978).

##### Phase 1

All participants made a coalition offer. In this offer, they (a) chose whom to send the offer, and (b) indicated how they would like to distribute the €100,000 between themselves and the chosen landowner in increments of €1,000.

##### Phase 2

Participants saw all offers that were made in Phase 1. They then selected one of the coalition offers that included them (made by themselves or another landowner).

##### Phase 3

Participants saw who selected which coalition offer. If two participants selected the same offer, the coalition would be formed and the payoffs were distributed as agreed. If no coalition was formed, a new round started in which participants went through the same three phases. This process was repeated until a coalition was formed.

#### Comprehension check

To gauge comprehension of the situation, participants completed a multiple choice quiz (correct answers in *italics*) asking for the amount of money the project developer would pay (*€100,000*/This depends on the size of the sold land), what the payoffs would be to the landowner not included in the coalition (This depends on the offer that was accepted/*This landowner doesn’t receive any money*), and which coalitions could be formed (AB & AC/AB & BC/AC & BC/*AB, AC, & BC*). If participants made a mistake, they were shown the correct answer. Participants could continue when answering the question correctly.

#### Dependent variables

To test our hypotheses, we focused on four dependent variables.

##### Formed coalition

As our main goal was replicating the Strength-is-Weakness effect, our main dependent variable was the formed coalition. That is, was the coalition formed an AB-, AC-, or BC-coalition?

##### Allocation in formed coalitions

For each formed coalition, we investigated whether those with more resources in a coalition attained a higher share of the payoffs. As allocations were made in increments of €1,000, participants made offers ranging from 1 to 100. Therefore, all offers and allocations are reported in the results without the three extra zeros.

##### First offer—Choice of bargaining partner

As an indirect measure of the expected offers made by the other bargainers, we analyzed to which other landowner participants make their first offer.

##### First offer—Allocation

To test whether the equity norm is applied in bargainers’ first offers, we analyzed the share of the payoffs bargainers claimed in their first offers.

### Results

#### Comprehension check

One participant falsely indicated that the size of the sold parcels would influence the size of the payoffs, two participants falsely indicated that the payoffs to the excluded landowner depended on the offer that was accepted, and 26 participants gave a wrong answer to the question which coalitions would be formed. As preregistered, we conducted analyses including all participants *and* including those that answered all questions correctly (*n* = 152). Only for two exploratory test did the interpretation between the two analyses differ (see Notes 5 and 6).

#### Formed coalitions

Replicating the Strength-is-Weakness effect, a chi-square goodness of fit test showed that BC-coalitions (*n* = 35; 67%) were formed more often than AC-coalitions (*n* = 15; 29%), and AB-coalitions (*n* = 2; 4%), χ^2^(2, *N* = 52) = 31.89, *p* < .001, *w* = 0.78. This difference remained significant when combining the AB- and AC-coalition and comparing them against the BC-coalition, χ^2^(1, *N* = 52) = 6.23, *p* = .01, *w* = 0.35. Translating these results into inclusion rates, A was only included in 19% of all coalitions, whereas B and C were included in 69% and 96%, respectively. See Table 1 for an overview of formed coalitions and allocations.

**Table 1.** Formed Coalitions and Mean Allocations for Each Position in Study 1.

Formed coalition	Allocation in Euro					
	*n*	%	*M*_A_	*M*_B_	*M*_C_	*SD*
AB	2	4	55.00	45.00	—	7.07
AC	15	29	54.13	—	45.87	3.91
BC	35	67	—	57.06	42.94	4.29

#### Allocation in formed coalitions

Because two coalition members allocated a fixed payoff of US$100,000, we measured inequality in payoffs by testing whether one of the two mean payoffs differed from US$50,000. One-sample *t* tests revealed that bargainers with more resources always obtained a larger share of the payoffs. In AC-coalitions, A obtained a larger share than C (*M*_A_ = 54.13, *SD* = 3.91) did, *t*(14) = 4.10, *p* = .001, *d* = 1.06, 95% CI_*d*_ = [−0.92, 3.04]. In BC-coalitions, B obtained a larger share than C (*M*_B_ = 57.06, *SD* = 4.29) did, *t*(34) = 9.73, *p* < .001, *d* = 1.65, 95% CI_*d*_ = [0.22, 3.07]. Finally, in AB-coalitions, A obtained a larger share than B (*M*_A_ = 55.00, *SD* = 7.07) did, but as only two AB-coalitions were formed, these numbers will not be interpreted.

#### First offers—Choice of bargaining partner

Chi-square goodness of fit tests showed that most bargainers sent a first offer to the weakest other bargainer, suggesting bargainers expect a higher payoff in a coalition with them. Landowner A made more first offers to C (*n* = 48) than to B (*n* = 10), χ^2^(1, *N* = 58) = 24.90, *p* < .001, *w* = 0.66. Likewise, Landowner B made more first offers to C (*n* = 56) than to A (*n* = 5), χ^2^(1, *N* = 61) = 42.64, *p* < .001, *w* = 0.84. Finally, Landowner C also made more first offers to B (*n* = 55) than to A (*n* = 6), χ^2^(1, *N* = 61) = 39.36, *p* < .001, *w* = 0.80. See Table 2 for an overview of proposed coalitions and mean proposed allocations for each position.

**Table 2.** Proposed Coalitions and Mean Proposed Allocations for Each Position in Study 1.

Position	Proposed coalition	Proposed allocation in Euro					
		*n*	%	*M*_A_	*M*_B_	*M*_C_	*SD*
A (4 acres)	AB	10	17.2	55.90	44.10	—	5.11
	AC	48	82.8	61.10	—	38.90	7.37
B (3 acres)	AB	5	8.2	55.40	44.60	—	3.65
	BC	56	91.8	—	58.21	41.79	4.55
C (2 acres)	AC	6	9.8	57.50	—	42.50	6.12
	BC	55	90.2	—	57.93	42.07	3.85

#### First offers—Allocation

**Figure 1.**
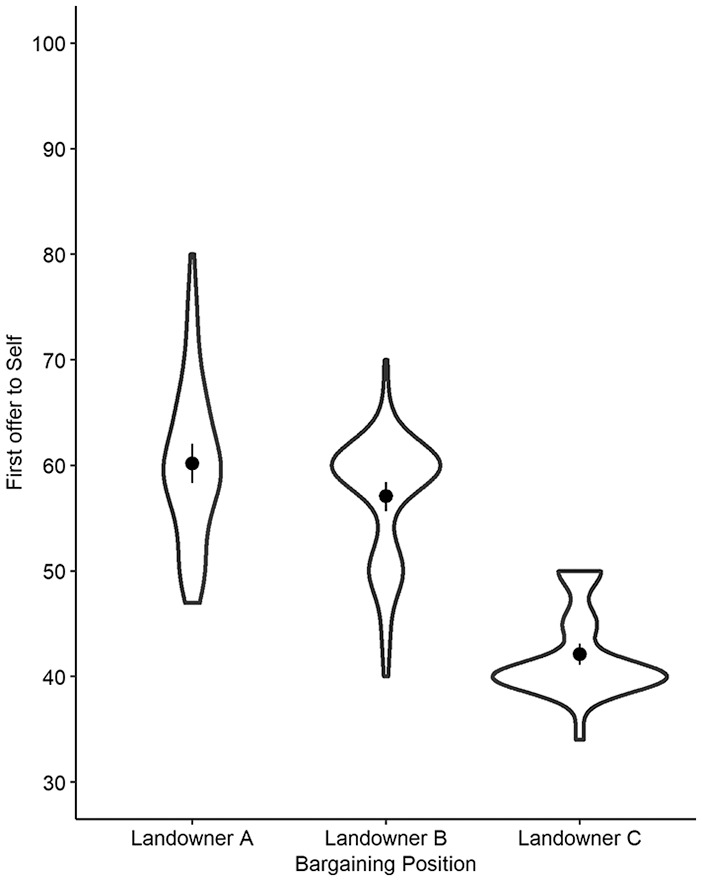
Violin plot of allocation to self by different bargaining positions in Study 1 with means (dot), CI^95^ (line), and probability density (width).

A one-way analysis of variance (ANOVA) showed that strong bargainers allocated more money to themselves in their first offers than weak bargainers did, *F*(2, 177) = 165.02, *p* < .001, η^2^ = .65, 95% CI_η2_ = [0.57, 0.71]. Tukey HSD (honest significance test) tests showed that Landowner A (*M* = 60.21, *SD* = 7.27) allocated more to themselves than Landowner B (*M* = 57.10, *SD* = 5.83), *p* = .012, *d* = 0.47, 95% CI_*d*_ = [0.10, 0.84], who in turn allocated more to themselves than Landowner C (*M* = 42.11, *SD* = 4.06), *p* < .001, *d* = 2.98, 95% CI_*d*_ = [2.46, 3.50].^4^ See Figure 1 for the distributions of allocations for the three bargaining positions.

#### The effect of first offers

We also explored the role of the magnitude of first offers in shaping the Strength-is-Weakness effect. As Bargainer C was almost always included in a coalition, and most first offers by A and B were made to Bargainer C, we compared the share of the payoffs claimed by Bargainers A and B in their first offers for each triad and analyzed whether this predicted the formation of strong or weak coalitions. As can be seen in Table 3, in most triads B made the most attractive offer, followed by situations in which A made a more attractive offer and finally situations in which A and B made identical offers. As can be seen, the Strength-is-Weakness effect occurred most often when B made the most attractive offer, or when offers were equally attractive, and least often when A made the most attractive offer, χ^2^(2, *N* = 52) = 8.26, *p* = .02, *w* = 0.40.^5^

**Table 3.** Attractiveness First Offers From Bargainers A and B and Inclusion of Strong Bargainer in Study 1.

Attractiveness first offers	Strong included	Strong excluded
A > B	9	6
A < B	7	19
A = B	1	10

### Discussion

In Study 1, we replicated the Strength-is-Weakness effect; the smallest BC-coalition was formed substantially more often than both the AB- and AC-coalitions. Landowner A was only included in 19% of the formed coalitions, whereas Landowners B and C were included in 69% and 96% of formed coalitions, respectively.

Participants’ first offers support the idea that the equity norm shapes the Strength-is-Weakness effect in two ways. First, individuals apply the equity norm; in their first offers strong bargainers demanded more payoffs than weak bargainers did. Moreover, the relative attractiveness of these first offers predicted which coalitions were formed, strengthening the idea that strong bargainers are often excluded due to their higher demands. Second, looking at the target of first offers it seems bargainers expected other bargainers to apply the equity norm. In their first offer, the majority of landowners preferred the weakest other bargainer. A plausible explanation for this choice is that bargainers expected the smallest coalition to be the most profitable coalition.

## Study 2

The goal of Study 2 was to see whether we could replicate the Strength-is-Weakness effect in a less controlled setting and using a different sample than undergraduate students. For this purpose, we recruited participants through Amazon Mechanical Turk. Previous comparisons between lab and Mechanical Turk samples seems to suggest no large differences in results (e.g., Arechar et al., 2018; Paolacci et al., 2010). However, research also seems to indicate that MTurkers are often non-naïve participants (Chandler et al., 2014). There seems to be evidence that the Strength-is-Weakness effect decreases with more exposure to coalition bargaining games (Kelley & Arrowood, 1960). Hence, there is a possibility that, due to prior exposure to other economic games, the Strength-is-Weakness effect would be less prominent in our Mechanical Turk sample. Study 2 was also the first online test of the Online Coalition Game allowing us to weigh the benefits and challenges of conducting coalition formation research on an online platform (see also Arechar et al., 2018).

### Method

Besides the few changes mentioned below, the materials and procedure were identical to those of Study 1, as were its hypotheses.

#### Participants and design

As preregistered, we aimed for 75 triads but obtained a sample of 80 triads (*N* = 240, *M*_age_ = 36.88 years, age range = 19–70, 119 females, 121 males). According to a sensitivity power analysis conducted in G*Power (Faul et al., 2007), this allowed us to detect a medium to large effect size (*w* = 0.35) when testing whether the distribution of formed coalitions differed from chance (i.e., equal proportions for all possible coalitions) with 80% power. Participants received US$2.40 for completing the hit and another US$0.05 cents per US$1,000 they attained in the scenario, leading to a payout of between US$2.40 and US$7.40. Within triads, participants were randomly assigned to one of the three positions in a 5(432) landowner game.

#### Materials and procedure

We made two changes compared with Study 1.

##### Matching procedure

In Study 2, we changed the matching procedure to address two specific challenges of running interactive studies online. First, online, participants starting the experiment but not finishing it are more prevalent than in the lab. As participants are interdependent once matched into a triad, this means that the dropout of one participant would lead to dropout of two matched participants. We assumed that this type of dropout would be most prevalent early in the study. By matching only participants who had already read most instructions, we minimized dropout of matched participants due to dropout of only one triad member.

Second, on Mechanical Turk we had no control over how many participants started the study at the same time. For this reason, if participants could not be matched within 5 min after they entered the matching screen, they were given the possibility to quit the study and collect their show-up fee. Moreover, we conducted the study in batches of between 30 and 45 participants to increase the odds that all participants would start playing around the same time and thus maximize the possibility that participants could be matched with other participants.

##### Timers

To make sure that matched idle participants did not stall their interaction partners, we added 2-min timers to the different pages in the interaction phase of the study. To minimize feelings of time pressure but still remind participants of the timer, these timers were only made visible after 1.5 min.

### Results

#### Comprehension check

Four participants falsely indicated that the size of the sold parcels would influence the size of the payoffs, 32 participants falsely indicated that the payoffs to the excluded landowner depended on the offer that was accepted, and 14 participants gave a wrong answer to the question which coalitions could be formed. Interpretations of all analyses did not differ when only including participants who have made no errors on the comprehension check (*n* = 199). We report analyses using all 240 participants.

#### Formed coalitions

Replicating the Strength-is-Weakness effect, a chi-square goodness of fit test showed that BC-coalitions (*n* = 52; 65%) were formed more often than AC-coalitions (*n* = 22; 27.5%) and AB-coalitions (*n* = 6; 7.5%), χ^2^(2, *N* = 80) = 40.90, *p* < .001, *w* = 0.72. This difference remained significant when combining the AB- and AC-coalition and comparing them against the BC-coalition, χ^2^(1, *N* = 80) = 7.20, *p* = .01, *w* = 0.30. Translating the results into inclusion rates, A was only included in 35% of all coalitions, whereas B and C were included in 72.5% and 92.5%, respectively. See Table 4 for an overview of formed coalitions and allocations.

**Table 4.** Formed Coalitions and Mean Allocations for Each Position in Study 2.

Formed coalition	Allocation in Euro					
	*n*	%	*M*_A_	*M*_B_	*M*_C_	*SD*
AB	6	7.5	52.50	47.50	—	2.74
AC	22	27.5	54.77	—	45.23	6.30
BC	52	65	—	55.10	44.90	6.60

#### Allocation in formed coalitions

As in Study 1, one-sample *t* tests showed that bargainers with more resources obtained a larger share of the payoffs (i.e., more than €50,000) in the coalitions that were formed. In AC-coalitions, A obtained a larger share than C (*M*_A_ = 54.77, *SD* = 6.30) did, *t*(21) = 3.55, *p* = .002, *d* = 0.76, 95% CI_*d*_ = [−1.88, 3.39]. In BC-coalitions, B obtained a larger share than C (*M*_B_ = 55.10, *SD* = 6.60) did, *t*(51) = 5.57, *p* < .001, *d* = 0.77, 95% CI_*d*_ = [−1.02, 2.57]. Finally, in AB-coalitions, A obtained a larger share than B (*M*_A_ = 52.50, *SD* = 2.74) did, but as only six AB-coalitions were formed; these numbers will not be interpreted.

#### First offers—Choice of bargaining partner

As in Study 1, Landowner A made more first offers to C (*n* = 66) than to B (*n* = 14), χ^2^(1, *N* = 80) = 33.80, *p* < .001, *w* = 0.65. Likewise, Landowner B made more first offers to C (*n* = 72) than to A (*n* = 8), χ^2^(1, *N* = 80) = 51.20, *p* < .001, *w* = 0.80. Finally, Landowner C made more first offers to B (*n* = 68) than to A (*n* = 12), χ^2^(1, *N* = 80) = 39.20, *p* < .001, *w* = 0.70. See Table 5 for an overview of proposed coalitions and mean proposed allocations for each position.

**Table 5.** Proposed Coalitions and Mean Proposed Allocations for Each Position in Study 2.

Position	Proposed coalition	Proposed allocation in Euro					
		*n*	%	*M*_A_	*M*_B_	*M*_C_	*SD*
A (4 acres)	AB	14	17.5	54.29	45.71	—	5.14
	AC	66	82.5	63.88	—	36.12	9.22
B (3 acres)	AB	8	10	50.37	49.63	—	9.16
	BC	72	90	—	55.83	44.17	6.76
C (2 acres)	AC	12	15	55.42	—	44.58	11.17
	BC	68	85	—	58.63	41.37	5.17

#### First offers—Allocation

**Figure 2.**
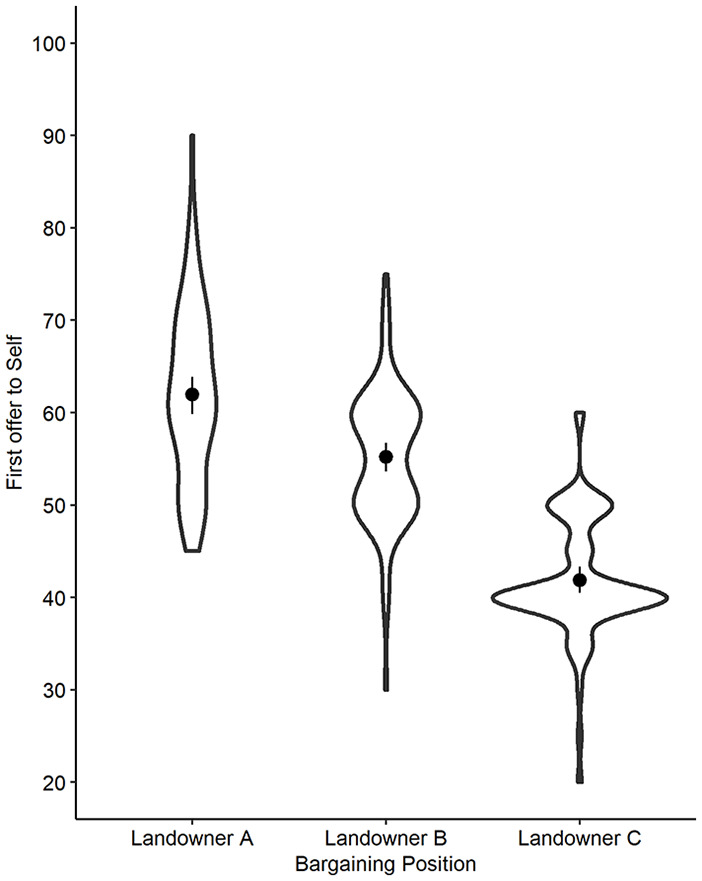
Violin plot of allocation to self by different bargaining positions in Study 2 with means (dot), CI^95^ (line), and probability density (width).

As in Study 1, a one-way ANOVA showed that strong bargainers allocated more money to themselves in their first offers, *F*(2, 237) = 139.22, *p* < .001, η^2^ = 0.54, 95% CI_η2_ = [0.27, 0.45]. Tukey HSD tests showed that Landowner A (*M* = 61.95, *SD* = 9.32) allocated more to themselves than Landowner B (*M* = 55.21, *SD* = 7.22), *p* < .001, *d* = 0.81, 95% CI_*d*_ = [0.48, 1.13], who in turn allocated more to themselves than Landowner C (*M* = 41.85, *SD* = 6.44), *p* < .001, *d* = 1.95, 95% CI_*d*_ = [1.57, 2.33]. See Figure 2 for the distributions of allocations for the three bargaining positions.

#### The effect of first offers

We again explored the role of the magnitude of first offers in shaping the Strength-is-Weakness effect. As can be seen in Table 6, in most triads B made the most attractive offer, followed by situations in which A made a more attractive offer and situations in which A and B made identical offers. Again, the Strength-is-Weakness effect occurred most often when B made the most attractive offer, or when offers were equally attractive, and least often when A made the most attractive offer, χ^2^(2, *N* = 80) = 10.44, *p* = .01, *w* = 0.36, 95% CI_*w*_ = [0.16, 0.59].

**Table 6.** Attractiveness First Offers From Bargainers A and B and Inclusion of Strong Bargainer in Study 2.

Attractiveness first offers	Strong included	Strong excluded
A > B	11	6
A < B	10	36
A = B	7	10

### Discussion

Study 2 again replicated the Strength-is-Weakness effect; the BC-coalition was formed substantially more often than the AB- and AC-coalitions. Landowner A was included in 15% of all formed coalitions, whereas B and C were included in 72.5% and 92.5%, respectively.

Moreover, first offers again provided support for both the application and the expectation of the equity norm as a mechanism underlying the Strength-is-Weakness effect. Strong bargainers claimed a higher share of the payoffs in their first offers than weaker bargainers did. The relative attractiveness of first offers also again predicted which coalitions were formed. First offers were again also predominately made toward weak bargainers rather than strong bargainers, which we take as indirect evidence that they expect the use of the equity norm and thus better allocations in smaller coalitions.

## Study 3

We designed Study 3 with two main goals in mind. First, we wanted a direct test of the assumption that bargainers anticipate more demanding offers from strong bargainers than from weak bargainers. Theoretically, expected use of the equity norm is assumed to lead to the formation of small coalitions (Gamson, 1961b, 1964). In Studies 1 and 2, we find that weak bargainers predominantly made offers to other weak bargainers, suggesting that they anticipated making a better deal with them. In Study 3, we wanted to substantiate this claim by directly asking participants about their expectations regarding the other bargainers’ offers.

Second, we wanted to assess (the antecedents of) the Strength-is-Weakness effect in a political setting rather than a landowner setting. Although we expected to replicate the Strength-is-Weakness effect in a political setting based on previous findings (Chertkoff, 1966) and theorizing (Warwick & Druckman, 2006), there are several differences between the two settings that one might suspect could influence the effect. One difference is that in political coalition formation, people might think the largest party deserves to be part of the coalition, something that is reflected in the notion that the largest party is often allowed to start negotiations (Bäck & Dumont, 2008). Another difference is that in our landowner setting, one might argue that participants might have formed a weak coalition to make sure the project developer did not get an extra acre of land for the same price. In governmental coalition formation, one could argue that parties might forgo this motivation to be efficient and, instead, form a larger coalition out of stability concerns. After all, when parties lose seats due to leaving politicians, a majority is more easily kept when this majority is larger to begin with. Moreover, although previous findings suggest that the Strength-is-Weakness effect is not particular to one particular configuration of resources (e.g., 5(432)), we employed a different configuration to further test the robustness of the effect. Finally, to test the effect in yet another population we recruited participants from Prolific.

To fulfill the above goals, participants engaged in a hypothetical 4(322) political scenario in which they were negotiators for either a strong party (with three seats) or for a weak party (with two seats). In this scenario, we asked participants to make a first offer, but also asked participants to estimate the first and final offers they would receive from the other two bargainers. Besides expecting to replicate the effects concerning first offers, we predicted that weak bargainers expected to be offered less by strong than by weak bargainers in both their first and final offers.

### Method

#### Participants and design

As preregistered, we obtained a sample of 320 participants (*M*_age_ = 40.10 years, age range = 19–77, 188 females, 133 males, two other), which according to sensitivity power analyses conducted in G*Power (Faul et al., 2007) allowed us to detect a small effect (*d_z_* = 0.19) when testing the hypotheses regarding expected offers with 80% power. Participants were randomly assigned to the strong (*n* = 160) and weak (*n* = 160) bargaining positions.

#### Materials and procedure

The instructions to participants were only slightly modified to match the hypothetical nature of the study. Participants learned that they were one of three individuals acting as a negotiator for one of three political parties in a newly founded municipality. They learned that Party A held three seats, Parties B and C both held two seats each and they needed to form a two-party coalition with at least four seats. The payoffs consisted of a £100 million budget that was to be allocated to the members of the coalition and participants were told that the party they negotiated for would pay them more if they were able to secure a larger share of the budget. Despite the fact that no actual bargaining took place, we also described the three steps of bargaining for maximum comparability.

##### Comprehension check

Participants completed the same three comprehension questions as in Studies 1 and 2. Afterward, participants received feedback on whether their answers were correct and were given the correct answers.

##### First offers

We asked participants to indicate to whom they would direct their first offer and how they would propose to allocate the £100 million between themselves and the target bargainer.

##### Expected first offers

We asked participants to estimate the other two bargainers would allocate the payoffs in a first offer to them.

##### Expected final offers

We asked participants to estimate how the other two bargainers would allocate the payoffs in a final offer to them.

### Results

#### Comprehension check

Nine participants falsely indicated that the size of the coalition would influence the size of the payoffs, 29 participants falsely indicated that the payoffs to the excluded party depended on the offer that was accepted, and 66 participants gave a wrong answer to the question which coalitions could be formed. Interpretations of all analyses did not differ when only including participants who made no errors on the comprehension check (*n* = 233). Below, we report analyses using all 320 participants.

#### First offers—Choice of bargaining partner

As both weak bargainers had two seats, strong bargainers had no way to differentiate between bargainers. Hence, we only conducted this analysis—and the analyses concerning expected offers—on weak bargainers. As hypothesized, and consistent with Studies 1 and 2, weak bargainers made more first offers to the other weak bargainer (*n* = 97) than to the strong bargainer (*n* = 63), χ^2^(1, *N* = 160) = 7.23, *p* =.01, *w* = 0.21.

#### First offers—Allocation

As hypothesized, an independent samples *t* test showed that strong bargainers (*M* = 61.87, *SD* = 10.26) allocated more money to themselves in their first offers than weak bargainers did (*M* = 51.44, *SD* = 10.57) did, *t*(318) = 8.95, *p* < .001, *d* = 1.00, 95% CI_*d*_ = [0.77, 1.23].

#### Expected first offers

As hypothesized, a paired samples *t* test showed that weak bargainers expected strong bargainers (*M* = 62.29, *SD* = 11.02) to allocate more money to themselves in their first offers than they expected weak bargainers to do (*M* = 52.65, *SD* = 9.05), *t*(159) = 11.12, *p* < .001, *d_z_* = 0.95, 95% CI_*dz*_ = [0.74, 1.15].

#### Expected final offers

As hypothesized, a paired samples *t* test showed that weak bargainers expected strong bargainers (*M* = 58.10, *SD* = 9.95) to allocate more money to themselves in their final offers than they expected weak bargainers (*M* = 50.28, *SD* = 7.47) to do, *t*(159) = 11.86, *p* < .001, *d_z_* = 0.86, 95% CI_*dz*_ = [0.70, 1.03].

### Discussion

In Study 3, we obtained additional evidence for the existence of the Strength-is-Weakness effect and the role of application and expectation of the equity norm in a 4(322) political setting. First, we again found that strong bargainers claimed a higher share of the payoffs in their first offers than weak bargainers did. Second, we again found that weak bargainers were more likely to make first offers to the other weak bargainer than to the strong bargainer. Finally, and most importantly, Study 3 provided direct evidence for the expected use of the equity norm by other bargainers: Weak bargainers expect more demanding claims from strong than from weak bargainers in their first and in their final offers.

## General Discussion

In three studies—two interactive and incentivized studies in a psychology undergraduate laboratory setting and on Amazon Mechanical Turk, and one scenario study using Prolific—we successfully replicated the Strength-is-Weakness effect in coalition formation (Caplow, 1956; Chaney & Vinacke, 1960; Gamson, 1964; Kelley & Arrowood, 1960; Murnighan, 1978a; van Beest et al., 2004a, 2011; Vinacke & Arkoff, 1957). We found the effect in different contexts (i.e., in the landowner paradigm and political setting), using different resource distributions (i.e., 5(432) and 4(322)), using different samples (i.e., undergraduate psychology students in a lab and Amazon Mechanical Turk and Prolific respondents), and regardless of whether the study is interactive and incentivized or hypothetical and nonincentivized.

### Mechanisms Underlying the Strength-Is-Weakness Effect

The three studies reported in this article also provide insights into the mechanisms underlying the Strength-is-Weakness effect. We have found considerable support for the assumption that the equity norm—the notion that those with more resources should obtain a higher share of the payoffs—shapes the Strength-is-Weakness effect (Gamson, 1961a, 1964; Komorita & Chertkoff, 1973). Importantly, we found that the equity norm shapes the effect in two distinct ways. First, bargainers apply the equity norm: Strong bargainers demand a higher share of the payoffs than weak bargainers do, making themselves less attractive as coalition partners. Second, bargainers expect others to use the equity norm: Expecting that strong bargainers make higher demands than weaker bargainers do, weak bargainers approach each other before even receiving a first offer from the strong bargainer. This suggests that actual first offers and expectations of first offer for a large part determine which coalitions are formed. Finally, the two pathways to the Strength-is-Weakness effect might interact. In Study 1 and Study 2, we find that strong bargainers are only included more often when they make *more* attractive offers than their weak counterpart does, not when they make *equally* attractive offers. This suggests that the expected use of the equity norm initially drives weak bargainer together and that weakest bargainers is only drawn to the strong bargainer when this expectation is substantially violated, when strong bargainers demand substantially less than they would when applying the equity norm.

Our findings also corroborate prior insights that both the confusion hypothesis (Kelley & Arrowood, 1960; Vinacke & Arkoff, 1957) and conspiracy theory (Wilke & Mulder, 1971, 1974) fail to provide plausible explanations for the Strength-is-Weakness effect. The main reason for this is that both proposed mechanisms place the cause of the effect at either only the (confused) strong bargainer or only the (conspiring) weak bargainers. Conversely, our results suggest that both strong and weak bargainers are responsible for the effect. Strong bargainers shape the effect because they apply the equity norm and therefore ask for a higher share of the payoffs. Weak bargainers shape the effect by avoiding strong bargainers because they expect them to apply the equity norm and thus claim a higher share of the payoffs.

The findings concerning the role of both the applied and expected use of the equity norm in shaping the Strength-is-Weakness effect provide hints for possible interventions to counteract the Strength-is-Weakness effect in situations in which it might be undesirable. First, knowing that the equity norm—and not the confusion hypothesis—underlies the effect, hints that interventions should be aimed at informing bargainers about the possible consequences of using the equity norm, rather than attempts at repairing incorrect perceptions regarding a link between resources and bargaining power. Second, our findings suggest that interventions should be aimed at both strong and weak bargainers, as they both seem to be responsible for the existence of the Strength-is-Weakness effect.

### Generalization of the Strength-Is-Weakness Effect

In this article, we have studied the Strength-is-Weakness effect in a setting in which the amount of resources held by a coalition did not determine the size of the payoffs (i.e., a simple situation; Komorita, 1984). An example of such a setting outside the lab is governmental coalition formation in which the number of ministerial posts (payoffs) to be distributed does not increase when a coalition with a higher number of seats (resources) is formed. Studies of coalition formation in West European democracies show use of the equity norm and exclusion of larger parties in these settings and thus the Strength-is-Weakness effect (Bäck & Dumont, 2008; Warwick, 1996).

Less is known about the (non)existence of the Strength-is-Weakness effect in settings in which having more resources leads to an increase of the payoffs (i.e., multivalued situations; Komorita, 1984). Arguably, this correlation between resources and payoffs would provide incentives to include the strong bargainer, leading to a decrease of the effect. A study by Komorita et al. (1989) shows that in such a setting strong bargainers are included more often when their relative strength in resources is not that large, but that they are not included more often when they have a lot more resources than the other bargainers. More research, however, is needed to test the robustness of this finding.

Another possible avenue for future research lies in how the availability of communication channels affects the Strength-is-Weakness effect. Studies by Swaab et al. (2009) showed that whether small coalitions (two parties) or large coalitions (three parties) are formed is determined by whether communication is computer-mediated or face-to-face and whether communication is private or open. Possibly, the Strength-is-Weakness effect would be most prevalent in private, computer-mediated channels. In these settings, Swaab and colleagues (2009) find that individuals become more self-focused, which potentially increases the use of self-serving use of the equity norm. Moreover, in these settings, weak bargainers might have an easier time excluding the strong bargainer than when they would have to defend this decision in an open communication channel. Of course, an open channel could also backfire if a strong bargainer makes an unattractive offer that drives all weak bargainers away simultaneously. Future research could investigate this by varying the communication channels available to bargainers in the 5(432) setting used in this article.

### Evaluating the Online Coalition Game

Studies 1 and 2 are the first implementations of the Online Coalition Game (Wissink et al., 2021). To determine whether this is a viable tool to conduct interactive online coalition research, it is important that effects are robust across platforms. Comparing key results between Studies 1 and 2, this seems to be the case. For example, there were no statistically significant differences between the studies conducted in our psychology lab and on Mechanical Turk in terms of formed coalition, χ^2^(2, *N* = 132) = 0.74, *p* = .69, *w* = 0.07, and allocation to self in first offers, *F*(1, 414) = 2.08, *p* = .15, *d* < .01, 95% CI_*d*_ = [−0.19, 0.20]. Moreover, the proportion of participants who did not make errors in our comprehension check did not differ substantially between our lab (84%) and Mechanical Turk (83%) sample. This suggests that differences in setting or sample characteristics (e.g., naivity; Chandler et al., 2014) do not affect the robustness of results obtained with the Online Coalition Game.

Another consideration when deciding to adopt a novel tool is weighing the challenges of adopting the new tool with its benefits. We argue that this is the case with the Online Coalition Game. The possibility of online bargaining on an online platform makes it possible to obtain a larger sample size in a shorter amount of time online compared with using a lab sample (a few hours spread across 3 days vs. 10 full-time workdays). The largest challenge we faced was the lower cost-efficiency online than in the lab. On Mechanical Turk we had to pay 35% of our sample the base fee because they could not be matched or because a matched participant dropped out. In the lab, we paid 13% of our sample that could not be matched.^6^ We do believe, however, that the benefit of having a large sample—and thus high-powered study—outweighs the extra costs.

## Conclusion

In the current article, we replicated the Strength-is-Weakness effect in three high-powered preregistered studies: two piece-rate incentivized studies using the novel Online Coalition Game (Studies 1 and 2) and one hypothetical study (Study 3). Across the three studies, we also investigated the mechanisms underlying the effect. Our results show support for the idea that the equity norm shapes the Strength-is-Weakness effect in two ways. Strong bargainers claimed a higher share of the payoffs in their first offers than weak bargainers do, making them less attractive coalition partners. In addition, weak bargainers expected strong bargainers to make these larger claims, directing weak bargainers to each other from the outset. Finally, the presented studies show that the Online Coalition Game is a viable tool for conducting high-powered coalition formation research, both in the lab and on online platforms.

## Notes

1. See https://aspredicted.org/dr9jy.pdf, https://aspredicted.org/98mg7.pdf, https://aspredicted.org/jk7hv.pdf

2. Note that bargaining theory (Komorita & Chertkoff, 1973) assumes that strong bargainers apply equity but weak bargainers apply equality and thus bargain for 50% of the payoffs. Regardless, this theory predicts that the weak coalition will be the cheapest winning coalition that will form at the expense of the strong bargainer.

3. We have not preregistered a prediction regarding the relative formation of AB- and AC-coalitions. However, based on the assumption that bargainers expect others to use the equity norm, it is to be expected that AC-coalitions are formed more often than AB-coalitions; both Bargainers A and B are most likely to make an offer to C, rather than to each other, making the AC-coalition more likely than the AB-coalition.

4. After excluding those who failed at least one of the comprehension check questions, Landowner A (*M* = 58.73, *SD* =6.52) did not significantly allocate more to themselves than Landowner B (*M* = 57.36, *SD* = 5.86), *p* = .44, *d* = 0.22, 95% CI_*d*_ = [−0.18, 0.62].

5. After excluding those who failed at least one of the comprehension check questions, we obtained the same pattern of results (Inclusion Rate of A: AttractiveOfferA = 50%, AttractiveOfferB = 23.1%, EquallyAttractiveOffers = 0%) but this was not statistically significant, χ^2^(2, *N* = 27) = 3.94, *p* = .13, *w* = 0.38. The size of the subsample suggests a lack of statistical power.

6. Note that these numbers might vary between labs (we typically have many students show up in our lab around the same time when all lectures end), but we might also improve our understanding of what times work best for online samples to reduce the number of people that could not be matched in time.
